# A multi-omics approach to solving problems in plant disease ecology

**DOI:** 10.1371/journal.pone.0237975

**Published:** 2020-09-22

**Authors:** Sharifa G. Crandall, Kaitlin M. Gold, María del Mar Jiménez-Gasco, Camila C. Filgueiras, Denis S. Willett

**Affiliations:** 1 Department of Plant Pathology and Environmental Microbiology, The Pennsylvania State University, University Park, PA, United States of America; 2 Plant Pathology & Plant Microbe Biology Section, Cornell AgriTech, Cornell University, Geneva, NY, United States of America; 3 Applied Chemical Ecology Technology, Cornell AgriTech, Cornell University, Geneva, NY, United States of America; Universita degli Studi di Pisa, ITALY

## Abstract

The swift rise of omics-approaches allows for investigating microbial diversity and plant-microbe interactions across diverse ecological communities and spatio-temporal scales. The environment, however, is rapidly changing. The introduction of invasive species and the effects of climate change have particular impact on emerging plant diseases and managing current epidemics. It is critical, therefore, to take a holistic approach to understand how and why pathogenesis occurs in order to effectively manage for diseases given the synergies of changing environmental conditions. A multi-omics approach allows for a detailed picture of plant-microbial interactions and can ultimately allow us to build predictive models for how microbes and plants will respond to stress under environmental change. This article is designed as a primer for those interested in integrating -omic approaches into their plant disease research. We review -omics technologies salient to pathology including metabolomics, genomics, metagenomics, volatilomics, and spectranomics, and present cases where multi-omics have been successfully used for plant disease ecology. We then discuss additional limitations and pitfalls to be wary of prior to conducting an integrated research project as well as provide information about promising future directions.

## Introduction

Plant disease ecology is inherently interdisciplinary and relies on the disparate fields of microbial ecology, epidemiology, plant physiology, and genetics to inform a critical observation: plants get sick. For more than a century, plant pathologists have observed that virulent microbes (e.g. bacteria, fungi, viruses, oomycetes) drive disease dynamics in susceptible plants given the right environmental conditions [1]. In natural systems, many plant pathogens have co-evolved antagonistic relationships with their hosts, and thus plant disease acts as an important force to regulate plant populations [2–4]. In managed systems, such as forest plantations or agro-ecosystems, plant health is important for maintaining yield and actively managing for plant diseases is a priority among land managers and farmers alike. Understanding and managing the ecology of these systems, however, has traditionally relied on a reductionist approach to research individual components (e.g., single or few microbe-plant interactions) related to specific disease pathologies rather than the complex ecological interactions between communities of microbes, hosts, and the environment.

**Fig 1:**
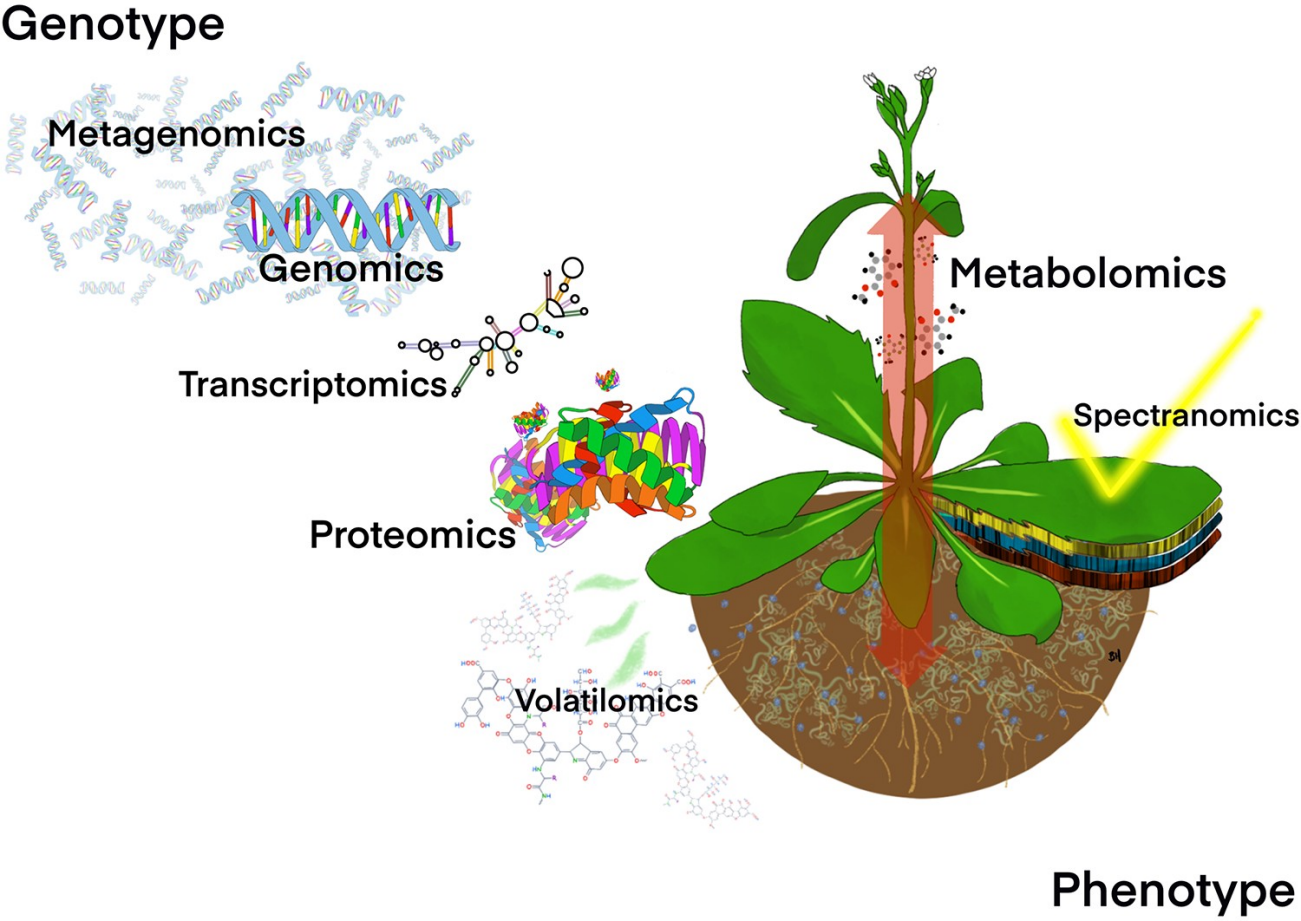
Multi-omics approaches inform the genotype to phenotype cascade. Omics approaches from the genotype (top left) to the phenotype (bottom right) inform plant disease ecology in a holistic manner and can shed light on microbial communities above and belowground. Red arrow on plant symbolizes dynamic intra-plant communication in the metabolome affecting the microbial community through released volatiles. Yellow check is light for spectranomics with multiple bands of sensing (below leaf).

Technological advances over the last few decades have enabled a new, more comprehensive approach to plant disease ecology allowing a holistic approach to identifying pathogenesis and its underlying mechanisms. Among these technologies are -omics tools that allow for the comprehensive examination of plant and microbial characteristics along the genotype-phenotype spectrum (Fig 1). Understanding these characteristics in a comprehensive fashion has facilitated discovery of mechanisms driving the ecology of plant defense and development of more effective management strategies ranging from improvements in plant breeding to field practices curtailing spread of plant pathogens.

These developments in omics-approaches to plant disease ecology have been particularly important in light of the current, rapidly changing environment. As increasing biotic and abiotic stressors impact plant health, adapting to and mitigating the effects of these stressors is critical for maintaining both healthy terrestrial ecosystems and productive agricultural systems. For example, globalization and the movement of people, plants, and microbes around the world can drastically change the composition and ecology of habitats. Invasive microbial pathogens and/or soilborne pathogens, especially those with broad host ranges, can cause the death of, in some cases, thousands of trees across a landscape [5, 6]. Both ecological invasions [7] and climate change are changing microbes and their environment [8]. For instance, shifting temperatures and the frequency and duration of weather conditions over time, result in phenomena such as the rapid evolution of microbial pathogens or environmental stress which can weaken plant hosts [8].

While some benefits from more holistic approaches have been realized, further opportunities exist through increasing integration of -omics technologies and platforms. Insights from genetics approaches can be tied to transcriptomics, metabolomics, and spectranomics to link multiple steps in the -omics cascade from genotype to phenotype. This integration, while still in its early stages, holds vast potential for unlocking fundamental and applied insights in microbial plant disease ecology. Understanding the genetic basis for plant pathogen susceptibility, how the plant microbial community influences expression, the cascading metabolomic and volatilomic pathways resulting from infection, and the spectranomic characteristics of foliage present numerous opportunities for intervention that can be tuned and optimized for plant disease management situations.

In this review, we discuss exactly these opportunities by beginning with an overview of -omics technologies including the unique challenges that each field faces. Next, we present recent case studies that highlight integration of multi-omics insights. We then offer additional opportunities and considerations for integrating these technologies and touch briefly on expected benefits resulting from this integration. These opportunities and considerations will be discussed in light of our principal goal: driving the development of healthy plants and their microbial communities in a constantly changing environment.

## The omics

### Genomics

The field of genomics, the study of the complete genetic makeup of organisms, has grown exponentially in the past 20 years since the milestone publication of the first draft of the human genome in 2001 [9, 10]. Although the word ‘genomics’ is somewhat recent [11], its origin dates back to the early 1900s when Johannsen introduced the concept of the gene and later when Hans Winkler coined the term genome in 1920. The first genomes sequenced were microbial, heralding an era of tool development and exponential generation of whole genome sequences: the bacterium *Haemophilus influenzae*, the first free-living organisms to have its whole genome sequenced via shotgun sequencing [12]; the fungus *Saccharomyces cerevisiae*, first eukaryote to have its whole genome sequenced [13]; and the nematode *Caenorhabditis elegans*, the first multicellular eukaryote [14]. Further development of Next Generation Sequencing (NGS) technologies has drastically reduced sequencing costs and accelerated the availability of whole genome sequences, *de novo* sequence assemblies and resequencing of multiple strains of a single species.

Microbial genomics is an interdisciplinary field that focuses on the structure, function, evolution, mapping, and editing of genomes for bacteria, fungi, archaea, viruses, and other microscopic organisms. Integration of genomics questions and tools can aid ecological questions, especially those that involve environmental change. For example, it is critical to understand the evolutionary history of the genome to understand if certain elements may change quickly under changing temperatures (rapid evolution). Sometimes, it may be necessary to draw comparisons across genomes to understand microbial functions in related species, or delve into transcriptomics and other –omics. Coupled with transcriptomics and proteomics, functional genomics uses genomic data to study gene and protein expression and function on a genome-wide or system-wide scale. The integration of genomics and transcriptomics has resulted in an increased understanding of various aspects related to plant pathogen biology, plant-pathogen interactions, and plant health [15, 16].

In investigations of plant disease ecology and plant health, it can be helpful to consider two main groups of microbial genomics. Structural genomics, focused on assigning and mapping genes and markers to individual chromosomes, results in a physical map of the whole genome. Functional genomics integrates genome sequences with an organism’s transcriptome (transcripts produced by a given organism) and proteome (encoded proteins) to describe gene functions and interactions [17, 18]. The combination of genome data and NGS-based RNA sequencing has significantly improved gene annotation and gene prediction [19]. However, there are many more “types” of genomics to be considered depending on the focus and context of research being done in contemporary plant disease ecology.

Comparative genomics aims to identify structural and functional genomic elements conserved within species or across different species [20]. Using this approach, we now know that the genome architecture of many fungal plant pathogens is highly diverse and dynamic, comprising regions of rapid evolution that can range from transposon-rich chromosome compartments to entire accessory chromosomes [21]. For example, by comparing the genomes of four *Fusarium* species clear genomic compartmentalization has been identified at both structural and functional levels: a core component of the genome that encodes functions necessary for growth and survival and is shared among *Fusarium* species, and an accessory component [22, 23]. The accessory genome comprises a variable number of small (<2Mb), supernumerary or conditionally dispensable chromosomes that are enriched for transposable elements, and carry what seem to be horizontally-transferred genes, some of which are known to be involved in plant pathogenicity. Similarly, *Verticillium* genomes differ in four regions of approximately 300–350 kb each, which are present in *V. dahliae* Ls.17 and absent in *V. alfalfae* Ms.102. These four regions contain genes that seem to vary considerably between strains and have functions putatively involved in pathogenicity and virulence of the pathogens [24, 25]. As more genomes are available and compared, we realize that these chromosomal dynamics are prevalent in many other plant pathogenic fungi [26–28].

In addition to elucidating genomic architecture, phylogenomics and population genomics address similar questions, but at different biological scales: relationships among species, and individuals and populations within species, respectively. The types of questions addressed include genome-wide evolutionary and demographic processes affecting population structure, evolutionary processes affecting speciation and the divergence of closely related taxa, and locus-specific effects, acting on specific genes or chromosomes that affect adaptation or defined phenotypes [26, 29, 30]. Such data provide a foundation for understanding the evolutionary potential of plant pathogens. One of the challenges we face with microbial genomics is the difficulty to establish and define species boundaries and population communities given the complexity of genome dynamics and the amount of observed horizontal gene transfer [31–34]. How these dynamics are affected by a changing environment in which we may see more host jumps and extreme environments is unpredictable [22, 33, 35].

### Metagenomics

Metagenomics builds on the tools and theories from molecular genomics to investigate DNA that is sourced directly from the environment. This field involves the extraction and amplification of microbial DNA targets from soil, water, air, plants and animals [36]. Although it is estimated that microbes are ubiquitous and found in every ecosystem on the planet, only approximately 1% of all microorganisms are culturable and identifiable using traditional first-generation, Sanger sequencing technologies [37]. The field of metagenomics grew out of the need for understanding the diversity and functional role of unculturable microbes. Microbiologists and plant pathologists first pioneered the techniques and tools in the late 1990s [38, 39].

Today the field has evolved to answer questions related to the ecological dynamics of microbial communities, multi-species regulation, mechanisms for microbe-microbe and host-microbe interactions, community coevolution [40]. The term metagenomics itself is comprised of the Greek prefix meta- which means to ‘transcend’ and describes how this discipline goes beyond traditional genomic techniques to identify the genomic diversity and gene function of microbes of what some scientists have coined unculturable microbial ‘dark matter’ [37]. With recent advances in high throughput DNA sequencing techniques, we are able to sequence either partial or the entire genome of microorganisms to answer questions such as microbial species composition, structure, phylogenetic relatedness, and function on the scale of entire microbial communities [41]. Today, a typical metagenomic pipeline involves the initial collection and processing of environmental DNA, the bioinformatic pre-processing of DNA sequence reads, determining the taxonomic profile as well as any functional or genomic elements of interest, statistical analyses, data validation, and finally visualization and communication of the results [42].

A major challenge in metagenomics is defining the microbial species concept itself. The biological species concept works well with more derived taxa (such as ourselves, *Homo sapiens*) where reproductive boundaries and barriers are strongly delineated. Bacteria, on the other hand, are currently assigned to a common species if their reciprocal, pairwise DNA re-association values are ≥ 70% during DNA hybridization experiments [43]. The common issue of microbial vertical transmission of DNA can complicate matters even further. Traditionally speaking, strains within a species must possess a certain degree of phenotypic consistency and species descriptions should be based on more than one type strain, such as a genetic variant or subtype. A microbial species name should technically only be assigned if its members can be distinguished from other species by at least one diagnostic phenotypic trait [43]. Of course it is difficult to assign microbial phenotypes to unculturable taxa. Another limitation for assigning taxonomic monikers is because of the lack of comprehensive public sequence databases available to map genetic reads to. Current databases such as GenBank and others such as Greengenes for bacteria and UNITE are great start, but still lack genomic information about the vast diversity of microbes still undiscovered on the planet.

In the case of plant health, metagenomic studies have revolved around two major categories to date. The first is for promoting plant growth through understanding endophytic microbes either within the shoots or roots communities [44]. The vast majority of these studies are still conducted with model plant organisms, such as maize, and there is a need to expand to other crops and into more natural systems. Others have ventured beyond the root into the rhizoplane and rhizosphere and use metagenomics to explore the role of functional genes for plant and soil health [45]. The second type of plant health research involves understanding how soil microbes suppress plant pathogens [46]. Again, the few studies conducted here are in crop pathosystems and there is a need to study the phenomenon of suppressive soils in other ecosystems, for example, managed and unmanaged forests and grasslands.

### Metabolomics

Metabolomics, the comprehensive profiling of all small molecules within an organism, is at the phenotypic end of the -omics spectrum and captures the results of the information cascade beginning with the genome and progressing through the transcriptome and proteome [47] (Fig 1). While metabolomics is firmly rooted in the chemical analysis of individual constituent compounds, the field has rapidly progressed in the past two decades to the ability to rapidly and exhaustively assess metabolites in multiple spatial and temporal dimensions [48–50]. While there are still limitations to attaining a complete understanding of the vast diversity of metabolites within a given organism or plant, targeted and untargeted approaches provide deep insights that, when coupled with complementary omics approaches, can yield an abundance of insights [51–53].

Metabolomics began in the 1970s with the expansion of medical analyses of compounds of interest using gas chromatography-mass spectrometry [54]. As technologies improved, medical profiling of human metabolites continued to expand for diagnostics and drug development. Applications of these profiling techniques in plants began in the 1990s with investigations into herbicide mode of action [55]. Linking functional genomics with metabolomics began in the late 1990s and has expanded in line with increasing technological capabilities in chromatography, mass spectrometry, and imaging technologies [54, 56]. Current metabolomics technology—typically ultra-high-pressure liquid chromatography coupled with high-resolution mass spectrometry or nuclear magnetic resonance spectroscopy—provides incredible resolution into the chemical phenotypes of study organisms able to capture and profile information on thousands of compounds [57–59].

These advances are not without limitations, however. A key challenge in metabolomics is the sheer diversity of potential metabolites present in any given sample. In contrast to micro-organisms or animals producing on the order of 1,500 and 2,500 unique metabolites respectively, a single plant species can produce upwards of 5,000 unique compounds [52]. All told, an estimated 200,000 unique metabolites are present in the plant kingdom [52, 60].

Targeted metabolomics approaches address these challenges by constraining profiling to a known set of annotated compounds [61]. Precise quantitative analysis of these compounds becomes possible through comparison to known libraries of analytical standards [62, 63]. While necessarily less than comprehensive, targeted approaches nonetheless can yield detailed information on a wide array of compounds numbering in the hundreds depending on the techniques used [62, 63]. Targeted metabolomics approaches are particularly useful for examining known processes and responses and allow for methodological optimization and improved resolution into biological processes of known importance.

Untargeted metabolomics approaches, in contrast, provide insight into unknowns [64]. Approaching the ideal of truly global metabolomics, untargeted metabolomics seeks to profile vast numbers of compounds present in biological samples through comparison of relative intensities [64]. In some cases, these compounds may be annotated and putatively identified [65]. Eschewing constraints allows broad comparisons to be made within and between experimental units for discovery of pathways and compounds driving biological processes [53]. Particularly important for multi-omics approaches, untargeted metabolomics facilitates connection discovery linking genomic, metagenomic, volatilomic, and spectromic approaches [52, 53].

### Volatilomics

Strictly speaking, volatilomics could be considered a subset of metabolomics [66]. However, the comprehensive profiling of high vapor pressure compounds released from organisms, volatilomics is much more than just a subset of metabolomics [67]. Where metabolomics seeks comprehensive profiling of predominantly intra-organisms compounds, volatilomics assesses those compounds released by an organism: the key components of chemically mediated inter-organismal communication [68]. Because volatilomics is concerned primarily with communication, it presents unique opportunities for understanding community dynamics in conjunction with a multi-omics approach.

As with metabolomics, plants use an astonishing array of compounds for communication ranging from plant hormones, terpenoids, green leaf volatiles, and volatiles produced and released upon attack from pests (herbivore-induced plant volatiles) [69]. These volatile compounds play numerous roles: influencing plant pollination, dispersal, herbivory and providing defenses against abiotic and biotic stress such as plant pathogens [68]. To date, it is estimated that plants alone release on the order of 30,000 different volatiles into both the atmosphere and rhizosphere [69]. Indeed, volatile communication is potentially even more important belowground where plant volatiles mediate everything from plant-pathogen interactions to plant-herbivore-natural enemy interactions in an environment where visual information processing is absent [70, 71].

The field of volatilomics grew out of the field of metabolomics as advances in gas chromatography coupled with mass spectrometry allowed for more in-depth profiling of gaseous volatile compounds [66]. Critical to the development of volatilomics as a field has been advances in collection methods of volatile organic compounds. To retain volatile compounds used in plant communication, headspace collection or sorptive materials are often used to capture volatiles in preparation for introduction to analysis by GC/MS [67]. These more modern approaches have begun to be used in high-throughput phenotyping were rapid assessment of plant volatilomic profiles are connected with gene by environment interactions in a non-destructive multi-omics approach [72]. Extending this approach to the plant-related microbiome holds the potential for discovery of new opportunities for identifying and controlling pests and pathogens [73, 74].

### Spectranomics

Foliar functional trait characterization has emerged in terrestrial ecology as a unifying concept to better understand both natural variability in vegetation function and variability in response to environmental change and stress. The idea of foliar functional traits has been enthusiastically embraced by the remote sensing community as many traits shown to strongly correlate with natural and stress-induced variation in plant function [75] can be detected and quantified from remotely sensed imagery [76–79]. Non-destructive, proximal and remote sensing of foliar functional traits with spectroscopy offers the capacity to fill gaps in space and time between labor intensive field measurements, reducing uncertainty in downstream analyses and decision making, as well as offering the ability to better evaluate hypotheses about plant function in response to abiotic and biotic stress. The use of spectroscopy combined with chemistry, taxonomy, and community ecology has been coined by leaders in the field as “spectranomics,” [80, 81]. The foundational components of the spectranomics approach is that 1) plants have chemical fingerprints that become increasingly unique when additional constituents are incorporated [77] and 2) spectroscopic signatures determine a portfolio of chemicals found in plants [82].

Only recently have plant pathologists begun to take advantage of the spectranomics trail blazed by terrestrial ecologists. Recent studies have helped establish in-situ (or foliar) and imaging spectroscopy (also known as “hyperspectral imaging”) as effective tools for early, non-destructive, and scalable biotic stress detection in natural and agroecosystems [83–86]. Both beneficial [87] and parasitic plant-microbe interactions [88] impact a variety of plant traits that can be non-destructively sensed. Microbes both directly and indirectly damage, impair, and/or alter foliar function, thus changing the chemical composition, such as through production of systemic effectors or secondary metabolites, or by physical presence of pathogen structures, such as hyphae and spores [89]. By taking a statistical approach to spectral data analysis, the sum total of the changes microbes impart to plant health can quantified with proximal and remote spectroscopy [83, 84]. This approach has been successful in increasing efficiency and accuracy in agronomic crop breeding pipelines [90–92]. Thus, spectral quantification of foliar functional traits allows us to detect, map, and model the biochemical and physiological pathosystem processes that engender our capacity to use spectroscopy for plant-microbe interaction sensing [86, 93–99].

Broadband and multispectral methods relying primarily on visible (VIS) and near-infrared (NIR) reflectance indices, such as normalized difference vegetation index (NDVI), have been used to sense late stage plant disease since the 1980s [100–103]. Changes in continuous, short wave infrared wavelengths (SWIR) have proved valuable for plant-microbe interaction sensing due to SWIR sensitivity to a range of foliar traits [104], including nutrient content [79, 105–107], water [108], photosynthetic capacity [109], physiology [110], phenolics and secondary metabolites [94, 111, 112], that are all impacted by plant microbe interactions. The advent of VSWIR (400-2500nm) sensing has reinvigorated the discipline of plant-microbe interaction sensing with its newly established capacity for robust pre-visual disease detection [94–99, 111, 113–116].

## Case studies in plant disease ecology under changing environments

### Multi-omics in plant defense: The salicylic acid pathway

Plant defence pathways mediate plant microbial interactions in changing environments regulating responses to abiotic and biotic stressors above and belowground [117–119]. A comprehensive understanding of the mechanisms underlying this ability and the nature of plant-mediated effects on microbial communities necessitates an integrated multi-omics approach. Nowhere is this as apparent as with the role of the salicylic acid (SA) signaling pathway. While there are many important plant defence pathways involving other critical plant hormones such as abscisic acid and jasmonic acid, for example, the SA pathway plays a critical role in regulating plant disease ecology with regulation of plant systemic acquired resistance and mediation of the interactions between members of plant microbial communities [120].

Understanding SA signaling began with early molecular studies as realization dawned that this phenolic secondary metabolite was nearly ubiquitous in the plant kingdom and involved in everything from plant reproduction and photosynthesis to mediating responses to abiotic and biotic stresses [121, 122]. These approaches began with metabolomics; interest in this signaling molecule drove research into networks of plant metabolites related to salicylic acid and their role as chemical signals [123, 124]. Initially, biochemical pathways involved in the production of SA were established picking up where the shikimic acid pathway left off and further developing through parallel isochorismate synthase and phenylalanine ammonia lyase pathways to production of the actual SA molecule which can be further modified downstream through addition of molecular tags such as methylation, glucosylatation, and conjugation with amino acids [123–125].

Following pathway elucidation, modern targeted and untargeted metabolomics approaches have characterized SA signaling cascades from plant stressors [126–128]. These metabolomics approaches have shed light not only on the manner in which plant responses are induced and effected, but also on SA induction cascades within the plant to affect other metabolic processes including cross-talk and regulation of other plant hormones [129, 130]. Particularly relevant for understanding microbial changes in the rhizosphere, metabolomics approaches are beginning to resolve how plant-microbe communication can shape microbial communities in changing environments [71]. Specifically, in the context of defense against plant pathogens, SA plays a role mediating pattern and effector triggered immune responses, producing of specific defense compounds ranging from anti-microbial peptides to terpenoids, programming death of infected and affected cells, and inducing systemic acquired resistance (SAR) [131–136].

While understanding of the SA pathway may have begun with metabolomics, it quickly progressed to inquiries into the genetic basis for observed phenoma, particularly related to systemic acquired resistance (SAR)—the ability of plants to acquire long-term resistance to pathogenic microbes [136, 137]. Genomic studies identified critical nonexpressor of pathogenesis (NPR) genes involved in SAR and how these genes can be systemically and transcriptionally programmed in situations where resistance is induced [138–140]. In addition to these NPR genes, attack by microbial pathogens and induction of the SA pathway can influence a wide range of gene expression regulating transcription networks that control plant defense responses involved in systemic acquired resistance [141, 142]. These genomics studies hold particular importance for plant breeding where desired genes encoding for SAR against pathogens could be bred into plants to ensure advantageous manipulation of microbial communities [143–145].

The SA signaling pathway and systemic acquired resistance not only affects the plant pathogen microbial community, but also a whole host of other organisms [120]. To do so, mobile volatile signals are used such as the methylated form of SA, methyl salicylate (MeSA) [146]. Volatilomics approaches to SA signaling have revealed that volatile profiles of plants change upon induction of the SA defense pathway [120]. These changes in volatile profiles can result in the induction of SA responses in neighboring plants and recruitment of natural enemies aboveground and belowground in the rhizosphere [147–150]. Exogenous induction of SA responses in perennial and annual plants can recruit entomopathogenic nematode natural enemies over long distances altering community structure and relationships with other nematodes and nematode microbial predators [151].

### Multi-omics for plant stress detection

The story of how these aspects of SA-regulated plant disease ecology were discovered is inextricably intertwined with the early history of multi-omics approaches. As integrated multi-omics has emerged, our resolution of the processes underlying SA plant defense signaling has improved and, with it, our ability to use this knowledge for positive intervention in managed natural systems. Early vegetative spectroscopic studies addressing the retrieval of foliar biochemicals primarily targeted pigments, nitrogen, protein, structural elements (cellulose and lignin) and mineral compounds [152, 153]. Soon after, this capacity was expanded to include phenolics and other classes of secondary metabolites associated with defense and the salicylic acid pathway [94, 111, 112]. In the past five years the boundaries of specificity in the characterizable trait range has expanded significantly to includes specific parameters related to photosynthesis such as maximum carboxylation and photosynthetic rates, sucrose, starch, fructose and free amino acids in leaves [91, 110, 154].

Spectroscopic methods such as fluorescence spectroscopy, Fourier-transform infrared spectroscopy, and chemometrics are used to better assess and understand plant-microbe interactions, but these methods require sampling and processing [155, 156]. The ability to quantify and predict plant metabolites from passive monitoring with vegetative spectroscopy, especially under field conditions, would enable in vivo metabolomics and thus opening the door to a new generation of plant, and plant-microbe, phenotyping approaches. In vivo metabolomics, with understandably less specificity than traditional metabolomics, with hyperspectral sensing and its value to precision agriculture has been hypothesized by multiple sub-groups in the plant sciences, and most recently by Martins et. al [157]. Pioneering work by Vergara-Diaz et al. [158] showed for the first time, the ability of hyperspectral field-sensors to non-destructively estimate foliar metabolite profiles. This work found that about one-quarter of metabolites detected in wheat leaves and ear bracts by GC-MS profiling could be satisfactorily predicted with hyperspectral data with validation accuracy over 50%. Many of the metabolites they could predict included sugars, amino acids and organic acids that play a central role in primary and secondary metabolism. Vergara-Diaz found the blue region proved to be the most relevant waveband for metabolite prediction regardless of the metabolite examined in both plant organs studied (leaf and ear). Likewise, these authors found consistency in their canopy models: the 1300–1400 and 2200–2400 nm regions were always the best determinants for metabolite prediction, and coincident with absorption bands known to be associated with sugars and nitrogen compounds.

Combining spectranomics and metabolomics would not be without its challenges: the assessment of plant biochemical traits at the canopy scale still faces considerable limitations from complexities related to canopy structure/density and the subsequent impact on light reflectance. Additionally, historical knowledge of the VIS-NIR-SWIR spectral features (spectral signals in plant reflectance) associated with plant metabolites is limited because typically other approaches have been used such as UV, MIR, X-ray, Raman or FTIR spectroscopy [155]. These more sensitive approaches can far more precisely quantify isolated metabolite concentrations, but do not account for how plant macro- and micro-physical properties impact how light interacts with these bonds. Prior knowledge of VIS-NIR-SWIR spectral features, such as those outlined in Curran [104] and Carter & Knapp [159], greatly aids in transferability and scaling, allowing us to make better sense of spectral data and the information it provides about underlying plant processes. Spectroscopic approaches sense genuine yet subtle changes to plant biochemistry, physiology, and morphology, all of which are impacted by plant-microbe interactions, and engender the origins our ability to use spectroscopic methods for plant sensing.

### Multi-omics in suppressive soils for plant disease management

The observation that soils have the capacity to suppress plant disease has been known for decades, some would argue for almost a century or longer in agricultural settings [160]. Yet understanding the specific soil biota involved, in what combination and under which environmental conditions elicit a suppressive response has eluded microbial ecologists [161]. Recently, with the advent of -omics approaches, we are beginning to better understand the ecology behind the suppressive effect [162]. Disease suppressive soils hold a subset of microbes, typically bacteria, that prevent the infection of a root by a plant pathogen or the development of the disease. Suppressive soils can act in either a general or specific manner. Broad soil suppression occurs when the supressive capabilities of broad microbial communities are harnessed, versus specific biocontrol which can occur using a single microbial taxon or a few taxa [163]. Understanding which taxa are involved in soil suppression using a metagenomics approach could reveal their various functions and shed light on the biological mechanism of suppression.

In order to apply basic knowledge of the soil microbial community identities and functions to address problems in the applied plant sciences such as conservation, restoration, forestry, and agriculture, it is essential to consider a multi-pronged approach. For instance, plant nutrient deficiencies have long been known to affect both crop yield, but also overall defense against pathogens. Using *Arabidopsis* as a model, researchers were able to tease apart the relationship between plant nutrient levels and host defense [164]. The authors found that the phosphate starvation response (PSR) plays an important role in shaping the root microbiome. The tools used to come to this result were a mix of amplicon-based sequencing to identify the host associated microbiome bacteria (16S) as well as genomic modeling and finally various metagenomic tests to reveal the functions of specific genes that modulated the response for the defense process [164]. The authors were able to conclude that certain beneficial microbial communities that help with plant defense against pathogens are shaped by the PSR.

Another application of a multi-omics approach is identifying more specific microbes that could be involved as biological control agents (BCA) against soilborne plant diseases and the creation of synthetic communities. Biocontrol research has typically taken a targeted approach that focuses on using a single or a few microbes to to suppress pathogenicity. With the development of high throughput sequencing techniques, such as amplicon-based sequencing, entire microbiomes were and are continuing to be identified. There is now need to shift away from community description toward functional understanding of these microbial communities in their capacity to not only suppress disease, but potentially have one microbe or a suite of microbes elicit antagonistic interactions to hinder plant disease [165, 166].

What is still needed for understanding the microbial ecology of soil suppression and the potential for biological applications to suppress plant disease are studies that seek predictive patterns and results. Specifically the spatial-temporal distribution of soil suppression and whether certain pathogens are consistently suppressed by specific taxa or functional groups of soil microbes. For instance certain sustainable land management practices such as the use of cover crops [167], organic cultivation [168, 169], or the use of diversified crop systems rather monocultures [170] have all been found to suppress plant diseases at varying levels of success depending on the crop pathogen, season, and climate. Decoupling microbial identity, function, pathogen identity, function, and environment are the next challenges for microbial ecologists—and doing so outside the lab and greenhouse to account for environmental factors is sorely needed.

### Frontiers in multi-omics for plant disease ecology

A promising area of -omics research is understanding the role of endophytes in plant disease. Endophytes are microbial organisms that live within plant tissue without causing visible symptoms and are essential to plant health and well being [171]. There is evidence for the critical role endophytes play in reducing herbivory both above and below ground [172], modulating plant immune response pathways and in some cases, systemic plant disease resistance [173], protecting from oxidative bursts and reduced gene expressions and remediating of abiotic and biotic plant through the specific downregulation of abscisic acid (ABA). Most research to date focuses on the function of one or a handful of endophytes rather than entire communities. One excellent study focused on the singular potato endophyte *Burkholderia phytofirmans* PsJN which should how various extra-cytoplasmatic functional group elements (sigma factors, group IV) were critical in facilitating other bacteria to sense changes in their surrounding environment such as temperature or moisture and shift their metabolic activity to survive the change. This paper used a dual -omic approach, by using high throughput sequencing to identify both the key player, *Burkholderia*, other bacteria, and basic metabolomics [174]. More recently, studies have been conducted to understand the diversity and structure of foliar endophytic fungi in forest ecosystems [175], however, there is still much to be understood about endophyte ecology in other systems and at the biome scale [176].

An additional promising frontier is the integration of spectranomics with genomics to construct multi-dimensional phylogenies that capture the evolutionary dynamics of leaf chemistry, structure, and more. Recent work by Meireles et al. showed that spectroscopy is capable of robustly capturing phylogenetic signals and that broad plant groups, orders, and families can be identified from reflectance spectra [177]. Using evolutionary models they found that different spectral regions evolved at different rates and under different constraint levels, mirroring the evolution of the underlying traits they correspond to. This breakthrough finding establishes that spectroscopy and spectranomics can provide novel insights into leaf evolution and plant phylogenetic diversity at scale. This approach could be applied to plant disease ecology to better understand the evolutionary dynamics of and history of beneficial plant microbe relationships as well as antagonistic.

## Promise, limitations & future directions

Integrating multi-omic approaches can help us understand who are the microbial players in the system, how they disperse and are distributed across time and space, and what are their individual and combined functions. As detailed in the case studies for this review, -omics can provide a richer picture of the mechanisms behind fundamental topics in plant disease ecology such as plant defence, stress response, and the potential for disease suppression. This is especially the case when compared to simply utilizing one -omic alone. These approaches, when integrated correctly at the right scale and for the right research question, promise to reveal a multi-dimensional view of plant disease. However, there are still many pitfalls and limitations toward integrating and using these approaches that researchers must be aware of before designing a research proposal and project. Below, we outline some of the promises as well as the pitfalls of -omics driven studies, as well as future directions for using these approaches for plant disease research.

When designing projects that could benefit from multi-omic approaches, there is a need to move away from strictly observational research questions to those that are hypothesis driven. Although the number of published research articles has grown exponentially in the past decade to reflect breakthroughs in high throughput sequencing, few have been applied to questions within the fields of environmental and conservation genomics that ask experimental questions [178]. A shift from hypotheses that measure correlation to those that decipher an underlying causation for an ecological phenomena would benefit the field of plant disease ecology. For example, there is a dire need to design hypothesis-based studies that test ecological theory in order to understand how microbial diversity and dynamics shift under a changing environment. There is merit for observational studies that describe the microbial players in a system; these studies allow a researcher to hone their ideas and questions once they explore the taxa present and certain environmental covariates. These initial studies often use predominately amplicon-based sequencing tools. While using amplicon-based sequencing to identify taxa is a valiant first-pass into defining the taxonomic and correlative boundaries of a system, descriptive studies can not reveal underlying mechanisms or drivers for the patterns observed. For instance, conducting ordinal statistical analyses to find patterns in microbial similarity in plant roots that are drought stressed versus those that are not can allow us to gain a better picture about microbial taxonomic presence or absence under environmental stress. Understanding why we see these patterns can not be gauged from strict observation. It is critical to understand the processes that drive the patterns we see with amplicon data, namely through designing and developing field, greenhouse, and laboratory experimental designs that elucidate community functional roles and dynamics and at different spatio-temporal scales [179, 180].

Another major limitation to conducting multi-omics are taxonomic or chemical compound databases that are currently in their nascent phases as more microbes are sequenced and discovered worldwide. Still, a handful of databases do exist that are well curated and are the standard for conducting -omics research. For both amplicon-based studies and metagenomic studies, open-source databases include RDP [181], Silva [182], Greengenes [183], and UNITE [184]. A Python-based tool that can be used to parse fungal operational taxonomic units (OTUs) by ecological guild independent of sequencing platform or analysis is FUNGuild [185]. FunFun is a novel database for fungal functional traits which is able to interface with other databases to explore and predict how fungal functional diversity varies by taxa, guild, and other evolutionary or ecological groups [186]. Common databases used in metabolomics and volatilomics studies include MassBank [187], Metabolomics Workbench [188], among other more specific databases [189]. Limitations in the scope and ability to use them as true reference databases for compound matching persist, however, with many researchers maintaining private instrument-specific libraries. Spectroscopy (spectranomics) databases are ecosml.org (Ecological Spectral Model Library) and ecosis.org (Ecological Spectral Information System) which are useful for finding spectral models and data on leaf nutrients, cellulose, and other physiological parameters.

Additional limitations to integrating multi-omics approaches in plant disease ecology include cost-per-sample (especially when considering multiple evaluation techniques) and difficulties in integrating disparate data-sets. -Omics information inherently comes in different formats with different means of preprocessing, analyzing, and interpreting the final results. Integrating those results across the genotype-phenotype spectrum can be challenging.

Addressing these limitations and realizing the potential of multi-omics research necessitates collaboration. Indeed, the motivation for writing this review was to spark such collaborations. No one person can be an expert across multi-omics domains; effectively conducting multi-omics research necessitates a integration of expertise just as it necessitates integration of datasets. This aspect of collaboration is perhaps the most promising feature of multi-omics research. Integrating a diversity of disciplines engenders an integration of perspectives. The challenges apparent in one discipline will likely benefit from fresh perspectives from another. Integrating these approaches will allow research collaborations to answer questions and address intractable problems that were previously not possible.

A multi-omics approach to solving problems in plant disease ecology has already led to breakthroughs in understanding plant defense, detecting plant stress, and managing disease with suppressive soils. This approach also seems poised to create breakthroughs in our understanding of endophytes. The promise of multi-omics in plant disease ecology extends beyond these areas, however, and looks to create breakthroughs in our understanding of how microbial communities respond in a changing environment for years to come [190].

## References

[1] Agrios G. Plant pathology 5th Edition: Elsevier Academic Press Burlington, Ma USA 2005; p. 79–103.

[2] Bever JD, Mangan SA, Alexander HM. Maintenance of plant species diversity by pathogens. Annual Review of Ecology, Evolution, and Systematics. 2015;46:305–325. 10.1146/annurev-ecolsys-112414-054306

[3] Burdon J. Fungal pathogens as selective forces in plant populations and communities. Australian Journal of Ecology. 1991;16(4):423–432. 10.1111/j.1442-9993.1991.tb01072.x

[4] Jeger M, Salama N, Shaw MW, Van Den Berg F, Van Den Bosch F. Effects of plant pathogens on population dynamics and community composition in grassland ecosystems: two case studies. European Journal of Plant Pathology. 2014;138(3):513–527. 10.1007/s10658-013-0325-1

[5] Rizzo D, Garbelotto M, Davidson J, Slaughter G, Koike S. *Phytophthora ramorum* as the cause of extensive mortality of *Quercus* spp. and *Lithocarpus densiflorus* in California. Plant Disease. 2002;86(3):205–214. 10.1094/PDIS.2002.86.3.205 30818595

[6] Dell B, Malajczuk N. Jarrah dieback—a disease caused by *Phytophthora cinnamomi* In: The Jarrah Forest. Springer; 1989 p. 67–87.

[7] Gilbert GS, Parker IM. Rapid evolution in a plant-pathogen interaction and the consequences for introduced host species. Evolutionary Applications. 2010;3(2):144–156. 10.1111/j.1752-4571.2009.00107.x 25567915 PMC3352484

[8] Santini A, Ghelardini L, et al Plant pathogen evolution and climate change. CABI Rev. 2015;10.

[9] Weissenbach J. The rise of genomics. Comptes rendus biologies. 2016;339(7-8):231–239. 10.1016/j.crvi.2016.05.002 27263360

[10] Lander ES, Linton LM, Birren B, Nusbaum C, Zody MC, Baldwin J, et al Initial sequencing and analysis of the human genome. 2001;409(6822):860–921. 10.1038/35057062 11237011

[11] McKusick VA, Ruddle FH. A new discipline, a new name, a new journal; 1987.

[12] Fleischmann RD, Adams MD, White O, Clayton RA, Kirkness EF, Kerlavage AR, et al Whole-genome random sequencing and assembly of *Haemophilus influenzae* Rd. Science. 1995;269(5223):496–512. 10.1126/science.7542800 7542800

[13] Goffeau A, Barrell BG, Bussey H, Davis R, Dujon B, Feldmann H, et al Life with 6000 genes. Science. 1996;274(5287):546–567. 10.1126/science.274.5287.546 8849441

[14] elegans Sequencing Consortium TC. Genome sequence of the nematode *C. elegans*: a platform for investigating biology. Science. 1998; p. 2012–2018. 10.1126/science.282.5396.2012 9851916

[15] Lindeberg M. Genome-enabled perspectives on the composition, evolution, and expression of virulence determinants in bacterial plant pathogens. Annual Review of Phytopathology. 2012;50:111–132. 10.1146/annurev-phyto-081211-173022 22559066

[16] Sundin GW, Wang N, Charkowski AO, Castiblanco LF, Jia H, Zhao Y. Perspectives on the transition from bacterial phytopathogen genomics studies to applications enhancing disease management: From promise to practice. Phytopathology. 2016;106(10):1071–1082. 10.1094/PHYTO-03-16-0117-FI 27183301

[17] Hieter P, Boguski M. Functional genomics: it’s all how you read it. Science. 1997;278(5338):601–602. 10.1126/science.278.5338.601 9381168

[18] Barone A, Chiusano ML, Ercolano MR, Giuliano G, Grandillo S, Frusciante L. Structural and functional genomics of tomato. International Journal of Plant Genomics. 2008;2008 10.1155/2008/820274 18317508 PMC2246074

[19] Yandell M, Ence D. A beginner’s guide to eukaryotic genome annotation. Nature Reviews Genetics. 2012;13(5):329–342. 10.1038/nrg3174 22510764

[20] Nobrega MA, Pennacchio LA. Comparative genomic analysis as a tool for biological discovery. The Journal of Physiology. 2004;554(1):31–39. 10.1113/jphysiol.2003.050948 14678488 PMC1664741

[21] Möller M, Stukenbrock EH. Evolution and genome architecture in fungal plant pathogens. Nature Reviews Microbiology. 2017;15(12):756 10.1038/nrmicro.2017.76 28781365

[22] Ma LJ, Van Der Does HC, Borkovich KA, Coleman JJ, Daboussi MJ, Di Pietro A, et al Comparative genomics reveals mobile pathogenicity chromosomes in *Fusarium*. Nature. 2010;464(7287):367–373. 10.1038/nature08850 20237561 PMC3048781

[23] Ma LJ, Geiser DM, Proctor RH, Rooney AP, O’Donnell K, Trail F, et al *Fusarium* pathogenomics. Annual Review of Microbiology. 2013;67:399–416. 10.1146/annurev-micro-092412-155650 24024636

[24] Klosterman SJ, Subbarao KV, Kang S, Veronese P, Gold SE, Thomma BP, et al Comparative genomics yields insights into niche adaptation of plant vascular wilt pathogens. PLoS Pathogens. 2011;7(7):e1002137 10.1371/journal.ppat.1002137 21829347 PMC3145793

[25] de Jonge R, Bolton MD, Kombrink A, van den Berg GC, Yadeta KA, Thomma BP. Extensive chromosomal reshuffling drives evolution of virulence in an asexual pathogen. Genome Research. 2013;23(8):1271–1282. 10.1101/gr.152660.112 23685541 PMC3730101

[26] Stukenbrock EH, Bataillon T, Dutheil JY, Hansen TT, Li R, Zala M, et al The making of a new pathogen: insights from comparative population genomics of the domesticated wheat pathogen *Mycosphaerella graminicola* and its wild sister species. Genome Research. 2011;21(12):2157–2166. 10.1101/gr.118851.110 21994252 PMC3227104

[27] Poppe S, Dorsheimer L, Happel P, Stukenbrock EH. Rapidly evolving genes are key players in host specialization and virulence of the fungal wheat pathogen *Zymoseptoria tritici* (*Mycosphaerella graminicola*). PLoS Pathogens. 2015;11(7):e1005055 10.1371/journal.ppat.1005055 26225424 PMC4520584

[28] Croll D, McDonald BA. The genetic basis of local adaptation for pathogenic fungi in agricultural ecosystems. Molecular Ecology. 2017;26(7):2027–2040. 10.1111/mec.13870 27696587

[29] de Vries S, Stukenbrock EH, Rose LE. Rapid evolution in plant–microbe interactions–an evolutionary genomics perspective. New Phytologist. 2020;226(5):1256–1262. 10.1111/nph.16458 31997351

[30] Grunwald S. Environmental soil-landscape modeling: Geographic information technologies and pedometrics. CRC Press; 2016.

[31] Ceresini PC, Castroagudín VL, Rodrigues FÁ, Rios JA, Aucique-Pérez CE, Moreira SI, et al Wheat blast: from its origins in South America to its emergence as a global threat. Molecular Plant Pathology. 2019;20(2):155–172. 10.1111/mpp.12747 30187616 PMC6637873

[32] Valent B, Farman M, Tosa Y, Begerow D, Fournier E, Gladieux P, et al *Pyricularia graminis-tritici* is not the correct species name for the wheat blast fungus: response to Ceresini et al.(MPP 20: 2). Molecular Plant Pathology. 2019;20(2):173 10.1111/mpp.12778 30697917 PMC6637902

[33] Aylward FO, Boeuf D, Mende DR, Wood-Charlson EM, Vislova A, Eppley JM, et al Diel cycling and long-term persistence of viruses in the ocean’s euphotic zone. Proceedings of the National Academy of Sciences. 2017;114(43):11446–11451. 10.1073/pnas.1714821114 PMC5663388 29073070

[34] VanInsberghe D, Arevalo P, Chien D, Polz MF. How can microbial population genomics inform community ecology? Philosophical Transactions of the Royal Society B. 2020;375(1798):20190253 10.1098/rstb.2019.0253 PMC7133533 32200748

[35] Friesen N, Fritsch RM, Blattner FR. Phylogeny and new intrageneric classification of *Allium* (Alliaceae) based on nuclear ribosomal DNA ITS sequences. Aliso: A Journal of Systematic and Evolutionary Botany. 2006;22(1):372–395. 10.5642/aliso.20062201.31

[36] Handelsman J, Tiedje J, Alvarez-Cohen L, Ashburner M, Cann IK, DeLong E, et al. Committee on metagenomics: challenges and functional applications; 2007.

[37] Solden L, Lloyd K, Wrighton K. The bright side of microbial dark matter: lessons learned from the uncultivated majority. Current Opinion in Microbiology. 2016;31:217–226. 10.1016/j.mib.2016.04.020 27196505

[38] Handelsman J, Rondon MR, Brady SF, Clardy J, Goodman RM. Molecular biological access to the chemistry of unknown soil microbes: a new frontier for natural products. Chemistry & Biology. 1998;5(10):R245–R249. 10.1016/S1074-5521(98)90108-9 9818143

[39] Chen K, Pachter L. Bioinformatics for whole-genome shotgun sequencing of microbial communities. PLoS Computational Biology. 2005;1(2):e24 10.1371/journal.pcbi.0010024 PMC1185649 16110337

[40] Segata N, Börnigen D, Morgan XC, Huttenhower C. PhyloPhlAn is a new method for improved phylogenetic and taxonomic placement of microbes. Nature Communications. 2013;4(1):1–11. 10.1038/ncomms3304 PMC3760377 23942190

[41] Caporaso JG, Lauber CL, Walters WA, Berg-Lyons D, Huntley J, Fierer N, et al Ultra-high-throughput microbial community analysis on the Illumina HiSeq and MiSeq platforms. The ISME journal. 2012;6(8):1621–1624. 10.1038/ismej.2012.8 22402401 PMC3400413

[42] Quince C, Walker AW, Simpson JT, Loman NJ, Segata N. Shotgun metagenomics, from sampling to analysis. Nature Biotechnology. 2017;35(9):833–844. 10.1038/nbt.3935 28898207

[43] Staley JT. The bacterial species dilemma and the genomic–phylogenetic species concept. Philosophical Transactions of the Royal Society B: Biological Sciences. 2006;361(1475):1899–1909. 10.1098/rstb.2006.1914 PMC1857736 17062409

[44] Fadiji AE, Babalola OO. Elucidating Mechanisms of Endophytes Used in Plant Protection and Other Bioactivities With Multifunctional Prospects. Frontiers in Bioengineering and Biotechnology. 2020;8:467 10.3389/fbioe.2020.00467 32500068 PMC7242734

[45] Yurgel ME, Kakad P, Zandawala M, Nässel DR, Godenschwege TA, Keene AC. A single pair of leucokinin neurons are modulated by feeding state and regulate sleep–metabolism interactions. PLoS Biology. 2019;17(2):e2006409 10.1371/journal.pbio.2006409 30759083 PMC6391015

[46] Larkin RP. Soil health paradigms and implications for disease management. Annual Review of Phytopathology. 2015;53:199–221. 10.1146/annurev-phyto-080614-120357 26002292

[47] Liu X, Locasale JW. Metabolomics: a primer. Trends in Biochemical Sciences. 2017;42(4):274–284. 10.1016/j.tibs.2017.01.004 28196646 PMC5376220

[48] Irie M, Fujimura Y, Yamato M, Miura D, Wariishi H. Integrated MALDI-MS imaging and LC–MS techniques for visualizing spatiotemporal metabolomic dynamics in a rat stroke model. Metabolomics. 2014;10(3):473–483. 10.1007/s11306-013-0588-8 24772057 PMC3984668

[49] Bartels B, Svatoš A. Spatially resolved in vivo plant metabolomics by laser ablation-based mass spectrometry imaging (MSI) techniques: LDI-MSI and LAESI. Frontiers in Plant Science. 2015;6:471 10.3389/fpls.2015.00471 26217345 PMC4498035

[50] Sumner LW, Yang DS, Bench BJ, Watson BS, Li C, Jones AD. Spatially resolved plant metabolomics Annual Plant Reviews online. 2018; p. 343–366.

[51] Dettmer K, Aronov PA, Hammock BD. Mass spectrometry-based metabolomics. Mass Spectrometry Reviews. 2007;26(1):51–78. 10.1002/mas.20108 16921475 PMC1904337

[52] Oksman-Caldentey KM, Saito K. Integrating genomics and metabolomics for engineering plant metabolic pathways. Current Opinion in Biotechnology. 2005;16(2):174–179. 10.1016/j.copbio.2005.02.007 15831383

[53] Johnson CH, Ivanisevic J, Siuzdak G. Metabolomics: beyond biomarkers and towards mechanisms. Nature Reviews Molecular Cell Biology. 2016;17(7):451–459. 10.1038/nrm.2016.25 26979502 PMC5729912

[54] Sumner LW, Mendes P, Dixon RA. Plant metabolomics: large-scale phytochemistry in the functional genomics era. Phytochemistry. 2003;62(6):817–836. 10.1016/S0031-9422(02)00708-2 12590110

[55] Sauter H, Lauer M, Fritsch H. Metabolic profiling of plants: a new diagnostic technique. ACS Publications; 1991.

[56] Oliver DJ, Nikolau B, Wurtele ES. Functional genomics: high-throughput mRNA, protein, and metabolite analyses. Metabolic Engineering. 2002;4(1):98–106. 10.1006/mben.2001.0212 11800579

[57] Ward JL, Baker JM, Beale MH. Recent applications of NMR spectroscopy in plant metabolomics. The FEBS journal. 2007;274(5):1126–1131. 10.1111/j.1742-4658.2007.05675.x 17298436

[58] Allwood JW, Ellis DI, Goodacre R. Metabolomic technologies and their application to the study of plants and plant–host interactions. Physiologia plantarum. 2008;132(2):117–135. 18251855 10.1111/j.1399-3054.2007.01001.x

[59] De Vos RC, Moco S, Lommen A, Keurentjes JJ, Bino RJ, Hall RD. Untargeted large-scale plant metabolomics using liquid chromatography coupled to mass spectrometry. Nature Protocols. 2007;2(4):778 10.1038/nprot.2007.95 17446877

[60] Hall R, Beale M, Fiehn O, Hardy N, Sumner L, Bino R. Plant metabolomics: the missing link in functional genomics strategies; 2002. 10.1105/tpc.140720 PMC543394 12119365

[61] Griffiths WJ, Koal T, Wang Y, Kohl M, Enot DP, Deigner HP. Targeted metabolomics for biomarker discovery. Angewandte Chemie International Edition. 2010;49(32):5426–5445. 10.1002/anie.200905579 20629054

[62] Roberts LD, Souza AL, Gerszten RE, Clish CB. Targeted metabolomics. Current Protocols in Molecular Biology. 2012;98(1):30–2. 10.1002/0471142727.mb3002s98 PMC3334318 22470063

[63] Lu W, Bennett BD, Rabinowitz JD. Analytical strategies for LC–MS-based targeted metabolomics. Journal of Chromatography B. 2008;871(2):236–242. 10.1016/j.jchromb.2008.04.031 PMC2607197 18502704

[64] Schrimpe-Rutledge AC, Codreanu SG, Sherrod SD, McLean JA. Untargeted metabolomics strategies—challenges and emerging directions. Journal of the American Society for Mass Spectrometry. 2016;27(12):1897–1905. 10.1007/s13361-016-1469-y 27624161 PMC5110944

[65] Dunn WB, Erban A, Weber RJ, Creek DJ, Brown M, Breitling R, et al Mass appeal: metabolite identification in mass spectrometry-focused untargeted metabolomics. Metabolomics. 2013;9(1):44–66. 10.1007/s11306-012-0434-4

[66] Cumeras R, Correig X. The Volatilome in Metabolomics In: Volatile Organic Compound Analysis in Biomedical Diagnosis Applications. Apple Academic Press; 2018 p. 23–50.

[67] Bicchi C, Maffei M. The plant volatilome: methods of analysis In: High-Throughput Phenotyping in Plants. Springer; 2012 p. 289–310. 10.1007/978-1-61779-995-2_15 22893295

[68] Rosenkranz M, Schnitzler JP. Plant volatiles. eLS. 2001; p. 1–9.

[69] Majchrzak T, Wojnowski W, Rutkowska M, Wasik A. Real-Time Volatilomics: A Novel Approach for Analyzing Biological Samples. Trends in Plant Science. 2020 10.1016/j.tplants.2019.12.005 31948793

[70] Insam H, Seewald MS. Volatile organic compounds (VOCs) in soils. Biology and Fertility of Soils. 2010;46(3):199–213. 10.1007/s00374-010-0442-3

[71] van Dam NM, Bouwmeester HJ. Metabolomics in the rhizosphere: tapping into belowground chemical communication. Trends in Plant Science. 2016;21(3):256–265. 10.1016/j.tplants.2016.01.008 26832948

[72] Jud W, Winkler JB, Niederbacher B, Niederbacher S, Schnitzler JP. Volatilomics: a non-invasive technique for screening plant phenotypic traits. Plant Methods. 2018;14(1):1–18. 10.1186/s13007-018-0378-4 30568721 PMC6297985

[73] Bailly A, Weisskopf L. Mining the volatilomes of plant-associated microbiota for new biocontrol solutions. Frontiers in Microbiology. 2017;8:1638 10.3389/fmicb.2017.01638 28890716 PMC5574903

[74] Parthasarathy S, Thiribhuvanamala G, Subramanian K, Paliyath G, Jayasankar S, Prabakar K. Volatile metabolites fingerprinting to discriminate the major post harvest diseases of mango caused by Colletotrichum gloeosporioides Penz. and Lasiodiplodia theobromae Pat. Annals of Phytomedicine. 2017;6(2):55–62.

[75] Wright IJ, Reich PB, Westoby M, Ackerly DD, Baruch Z, Bongers F, et al The worldwide leaf economics spectrum. Nature. 2004;428(6985):821–827. 10.1038/nature02403 15103368

[76] Townsend PA, Foster JR, Chastain RA, Currie WS. Application of imaging spectroscopy to mapping canopy nitrogen in the forests of the central Appalachian Mountains using Hyperion and AVIRIS. IEEE Transactions on Geoscience and Remote Sensing. 2003;41(6):1347–1354. 10.1109/TGRS.2003.813205

[77] Ustin SL, Roberts DA, Gamon JA, Asner GP, Green RO. Using imaging spectroscopy to study ecosystem processes and properties. Bioscience. 2004;54(6):523–534. 10.1641/0006-3568(2004)054[0523:UISTSE]2.0.CO;2

[78] Ustin SL, Gamon JA. Remote sensing of plant functional types. New Phytologist. 2010;186(4):795–816. 10.1111/j.1469-8137.2010.03284.x 20569415

[79] Singh A, Serbin SP, McNeil BE, Kingdon CC, Townsend PA. Imaging spectroscopy algorithms for mapping canopy foliar chemical and morphological traits and their uncertainties. Ecological Applications. 2015;25(8):2180–2197. 10.1890/14-2098.1 26910948

[80] Asner GP, Martin RE. Airborne spectranomics: mapping canopy chemical and taxonomic diversity in tropical forests. Frontiers in Ecology and the Environment. 2009;7(5):269–276. 10.1890/070152

[81] Asner GP, Martin RE. Spectranomics: Emerging science and conservation opportunities at the interface of biodiversity and remote sensing. Global Ecology and Conservation. 2016;8:212–219. 10.1016/j.gecco.2016.09.010

[82] Jacquemoud S, Verdebout J, Schmuck G, Andreoli G, Hosgood B. Investigation of leaf biochemistry by statistics. Remote Sensing of Environment. 1995;54(3):180–188. 10.1016/0034-4257(95)00170-0

[83] Mahlein AK. Plant disease detection by imaging sensors–parallels and specific demands for precision agriculture and plant phenotyping. Plant Disease. 2016;100(2):241–251. 10.1094/PDIS-03-15-0340-FE 30694129

[84] Mahlein AK, Kuska MT, Behmann J, Polder G, Walter A. Hyperspectral sensors and imaging technologies in phytopathology: state of the art. Annual Review of Phytopathology. 2018;56:535–558. 10.1146/annurev-phyto-080417-050100 30149790

[85] Zarco-Tejada P, Camino C, Beck P, Calderon R, Hornero A, Hernández-Clemente R, et al Previsual symptoms of *Xylella fastidiosa* infection revealed in spectral plant-trait alterations. Nature Plants. 2018;4(7):432–439. 10.1038/s41477-018-0189-7 29942047

[86] Fallon B, Yang A, Lapadat C, Armour I, Juzwik J, Montgomery RA, et al Spectral differentiation of oak wilt from foliar fungal disease and drought is correlated with physiological changes. Tree Physiology. 2020;40(3):377–390. 10.1093/treephys/tpaa005 32031662

[87] Fisher JB, Sweeney S, Brzostek ER, Evans TP, Johnson DJ, Myers JA, et al Tree-mycorrhizal associations detected remotely from canopy spectral properties. Global Change Biology. 2016;22(7):2596–2607. 10.1111/gcb.13264 27282323

[88] Mahlein AK, Kuska MT, Thomas S, Wahabzada M, Behmann J, Rascher U, et al Quantitative and qualitative phenotyping of disease resistance of crops by hyperspectral sensors: seamless interlocking of phytopathology, sensors, and machine learning is needed! Current Opinion in Plant Biology. 2019;50:156–162. 10.1016/j.pbi.2019.06.007 31387067

[89] Agrios G. Plant pathogens and disease: general introduction. 2009.

[90] Ge Y, Atefi A, Zhang H, Miao C, Ramamurthy RK, Sigmon B, et al High-throughput analysis of leaf physiological and chemical traits with VIS–NIR–SWIR spectroscopy: a case study with a maize diversity panel. Plant Methods. 2019;15(1):66 10.1186/s13007-019-0450-8 31391863 PMC6595573

[91] Meacham-Hensold K, Montes CM, Wu J, Guan K, Fu P, Ainsworth EA, et al High-throughput field phenotyping using hyperspectral reflectance and partial least squares regression (PLSR) reveals genetic modifications to photosynthetic capacity. Remote Sensing of Environment. 2019;231:111176 10.1016/j.rse.2019.04.029 31534277 PMC6737918

[92] Meacham-Hensold K, Fu P, Wu J, Serbin S, Montes CM, Ainsworth E, et al Plot-level rapid screening for photosynthetic parameters using proximal hyperspectral imaging. Journal of Experimental Botany. 2020;71(7):2312–2328. 10.1093/jxb/eraa068 32092145 PMC7134947

[93] Arens N, Backhaus A, Döll S, Fischer S, Seiffert U, Mock HP. Non-invasive presymptomatic detection of *Cercospora beticola* infection and identification of early metabolic responses in sugar beet. Frontiers in Plant Science. 2016;7:1377 10.3389/fpls.2016.01377 27713750 PMC5031787

[94] Couture J, Singh A, Charkowski A, Groves R, Gray S, Bethke P, et al Integrating spectroscopy with potato disease management. Plant Disease. 2018;102(11):2233–2240. 10.1094/PDIS-01-18-0054-RE 30145947

[95] Fallon B, Yang A, Nguyen C, Armour I, Juzwik J, Montgomery RA, et al. Leaf and canopy spectra, symptom progression, and physiological data from experimental detection of oak wilt in oak seedlings. Experimental Dataset, University of Minnesota. 2019; 10.13020/cgy7-2564.

[96] Gold KM, Townsend PA, Herrmann I, Gevens AJ. Investigating potato late blight physiological differences across potato cultivars with spectroscopy and machine learning. Plant Science. 2019; p. 110316 32534618 10.1016/j.plantsci.2019.110316

[97] Gold KM, Gevens AJ, Townsend PA. System for Detection of Disease in Plants. US Patent App 16/251,415. 2019.

[98] Gold KM, Townsend PA, Larson ER, Herrmann I, Gevens AJ. Contact Reflectance Spectroscopy for Rapid, Accurate, and Nondestructive *Phytophthora infestans* Clonal Lineage Discrimination. Phytopathology. 2020;110(4):851–862. 10.1094/PHYTO-08-19-0294-R 31880984

[99] Gold KM, Townsend PA, Chlus A, Herrmann I, Couture JJ, Larson ER, et al Hyperspectral measurements enable pre-symptomatic detection and differentiation of contrasting physiological effects of late blight and early blight in potato. Remote Sensing. 2020;12(2):286 10.3390/rs12020286

[100] Hatfield P, Pinter P Jr. Remote sensing for crop protection. Crop Protection. 1993;12(6):403–413. 10.1016/0261-2194(93)90001-Y

[101] Nagarajan S, Seibold G, Kranza J, Saari E, Joshi L. Monitoring wheat rust epidemics with the Landsat-2 satellite. Phytopathology. 1984;74(5):585–587. 10.1094/Phyto-74-585

[102] Jackson R. Remote sensing of biotic and abiotic plant stress. Annual Review of Phytopathology. 1986;24(1):265–287. 10.1146/annurev.py.24.090186.001405

[103] Nilsson HE. Remote sensing and image analysis in plant pathology. Canadian Journal of Plant Pathology. 1995;17(2):154–166. 10.1080/07060669509500707 18999971

[104] Curran PJ. Remote sensing of foliar chemistry. Remote Sensing of Environment. 1989;30(3):271–278. 10.1016/0034-4257(89)90069-2

[105] Gillon D, Houssard C, Joffre R. Using near-infrared reflectance spectroscopy to predict carbon, nitrogen and phosphorus content in heterogeneous plant material. Oecologia. 1999;118(2):173–182. 10.1007/s004420050716 28307692

[106] Serbin SP, Dillaway DN, Kruger EL, Townsend PA. Leaf optical properties reflect variation in photosynthetic metabolism and its sensitivity to temperature. Journal of Experimental Botany. 2012;63(1):489–502. 10.1093/jxb/err294 21984647 PMC3245480

[107] Zhai S, Chen H, Ding C, Zhao X. Double-negative acoustic metamaterial based on meta-molecule. Journal of Physics D: Applied Physics. 2013;46(47):475105 10.1088/0022-3727/46/47/475105

[108] Gao BC. NDWI—A normalized difference water index for remote sensing of vegetation liquid water from space. Remote Sensing of Environment. 1996;58(3):257–266. 10.1016/S0034-4257(96)00067-3

[109] Oren R, Schulze ED, Matyssek R, Zimmermann R. Estimating photosynthetic rate and annual carbon gain in conifers from specific leaf weight and leaf biomass. Oecologia. 1986;70(2):187–193. 10.1007/BF00379238 28311656

[110] Serbin SP, Wu J, Ely KS, Kruger EL, Townsend PA, Meng R, et al From the Arctic to the tropics: multibiome prediction of leaf mass per area using leaf reflectance. New Phytologist. 2019;224(4):1557–1568. 10.1111/nph.16123 31418863

[111] Couture JJ, Singh A, Rubert-Nason KF, Serbin SP, Lindroth RL, Townsend PA. Spectroscopic determination of ecologically relevant plant secondary metabolites. Methods in Ecology and Evolution. 2016;7(11):1402–1412. 10.1111/2041-210X.12596

[112] Kokaly RF, Skidmore AK. Plant phenolics and absorption features in vegetation reflectance spectra near 1.66 *μ*m. International Journal of Applied Earth Observation and Geoinformation. 2015;43:55–83. 10.1016/j.jag.2015.01.010

[113] Wu D, Feng L, Zhang C, He Y. Early detection of *Botrytis cinerea* on eggplant leaves based on visible and near-infrared spectroscopy. Transactions of the ASABE. 2008;51(3):1133–1139. 10.13031/2013.24504

[114] Rumpf T, Mahlein AK, Steiner U, Oerke EC, Dehne HW, Plümer L. Early detection and classification of plant diseases with support vector machines based on hyperspectral reflectance. Computers and Electronics in Agriculture. 2010;74(1):91–99. 10.1016/j.compag.2010.06.009

[115] Xie C, Yang C, He Y. Hyperspectral imaging for classification of healthy and gray mold diseased tomato leaves with different infection severities. Computers and Electronics in Agriculture. 2017;135:154–162. 10.1016/j.compag.2016.12.015

[116] Bienkowski D, Aitkenhead MJ, Lees AK, Gallagher C, Neilson R. Detection and differentiation between potato (*Solanum tuberosum*) diseases using calibration models trained with non-imaging spectrometry data. Computers and Electronics in Agriculture. 2019;167:105056 10.1016/j.compag.2019.105056

[117] Stout MJ, Thaler JS, Thomma BP. Plant-mediated interactions between pathogenic microorganisms and herbivorous arthropods. Annu Rev Entomol. 2006;51:663–689. 10.1146/annurev.ento.51.110104.151117 16332227

[118] Bezemer TM, van Dam NM. Linking aboveground and belowground interactions via induced plant defenses. Trends in Ecology & Evolution. 2005;20(11):617–624. 10.1016/j.tree.2005.08.006 16701445

[119] Kumar D, Chapagai D, Dean P, Davenport M. Biotic and abiotic stress signaling mediated by salicylic acid In: Elucidation of Abiotic Stress Signaling in Plants. Springer; 2015 p. 329–346.

[120] Filgueiras CC, Martins AD, Pereira RV, Willett DS. The Ecology of Salicylic Acid Signaling: Primary, Secondary and Tertiary Effects with Applications in Agriculture. International Journal of Molecular Sciences. 2019;20(23):5851 10.3390/ijms20235851 PMC6928651 31766518

[121] Mahdi J, Mahdi A, Mahdi A, Bowen I. The historical analysis of aspirin discovery, its relation to the willow tree and antiproliferative and anticancer potential. Cell Proliferation. 2006;39(2):147–155. 10.1111/j.1365-2184.2006.00377.x 16542349 PMC6496865

[122] Raskin I. Role of salicylic acid in plants. Annual Review of Plant Biology. 1992;43(1):439–463. 10.1146/annurev.pp.43.060192.002255

[123] Dempsey DA, Vlot AC, Wildermuth MC, Klessig DF. Salicylic acid biosynthesis and metabolism. The *Arabidopsis* book/American Society of Plant Biologists. 2011;9. 10.1199/tab.0156 PMC3268552 22303280

[124] Chen Z, Zheng Z, Huang J, Lai Z, Fan B. Biosynthesis of salicylic acid in plants. Plant Signaling & Behavior. 2009;4(6):493–496. 10.4161/psb.4.6.8392 19816125 PMC2688294

[125] Kumar D, Haq I, Chapagai D, Tripathi D, Donald D, Hossain M, et al Hormone signaling: current perspectives on the roles of salicylic acid and its derivatives in plants In: The Formation, Structure and Activity of Phytochemicals. Springer; 2015 p. 115–136.

[126] Noctor G, Lelarge-Trouverie C, Mhamdi A. The metabolomics of oxidative stress. Phytochemistry. 2015;112:33–53. 10.1016/j.phytochem.2014.09.002 25306398

[127] Mhlongo M, Tugizimana F, Piater L, Steenkamp PA, Madala N, Dubery I. Untargeted metabolomics analysis reveals dynamic changes in azelaic acid-and salicylic acid derivatives in LPS-treated Nicotiana tabacum cells. Biochemical and biophysical research communications. 2017;482(4):1498–1503. 10.1016/j.bbrc.2016.12.063 27956183

[128] Mhlongo MI, Piater LA, Madala NE, Labuschagne N, Dubery IA. The chemistry of plant–microbe interactions in the rhizosphere and the potential for metabolomics to reveal signaling related to defense priming and induced systemic resistance. Frontiers in Plant Science. 2018;9:112 10.3389/fpls.2018.00112 29479360 PMC5811519

[129] Caarls L, Pieterse CM, Van Wees S. How salicylic acid takes transcriptional control over jasmonic acid signaling. Frontiers in Plant Science. 2015;6:170 10.3389/fpls.2015.00170 25859250 PMC4373269

[130] Kunkel BN, Brooks DM. Cross talk between signaling pathways in pathogen defense. Current Opinion in Plant Biology. 2002;5(4):325–331. 10.1016/S1369-5266(02)00275-3 12179966

[131] Tsuda K, Katagiri F. Comparing signaling mechanisms engaged in pattern-triggered and effector-triggered immunity. Current Opinion in Plant Biology. 2010;13(4):459–465. 10.1016/j.pbi.2010.04.006 20471306

[132] Sudisha J, Sharathchandra R, Amruthesh K, Kumar A, Shetty HS. Pathogenesis related proteins in plant defense response In: Plant defence: biological control. Springer; 2012 p. 379–403.

[133] Heath MC. Hypersensitive response-related death In: Programmed cell death in higher plants. Springer; 2000 p. 77–90.

[134] Klessig DF, Choi HW, Dempsey DA. Systemic acquired resistance and salicylic acid: past, present, and future. Molecular Plant-Microbe Interactions. 2018;31(9):871–888. 10.1094/MPMI-03-18-0067-CR 29781762

[135] Lu H, Greenberg JT, Holuigue L. Salicylic acid signaling networks. Frontiers in Plant Science. 2016;7:238 10.3389/fpls.2016.00238 26941775 PMC4764731

[136] Gaffney T, Friedrich L, Vernooij B, Negrotto D, Nye G, Uknes S, et al Requirement of salicylic acid for the induction of systemic acquired resistance. Science. 1993;261(5122):754–756. 10.1126/science.261.5122.754 17757215

[137] Durrant WE, Dong X. Systemic acquired resistance. Annu Rev Phytopathol. 2004;42:185–209. 10.1146/annurev.phyto.42.040803.140421 15283665

[138] Innes R. The positives and negatives of NPR: a unifying model for salicylic acid signaling in plants. Cell. 2018;173(6):1314–1315. 10.1016/j.cell.2018.05.034 29856948

[139] Fu ZQ, Yan S, Saleh A, Wang W, Ruble J, Oka N, et al NPR3 and NPR4 are receptors for the immune signal salicylic acid in plants. Nature. 2012;486(7402):228–232. 10.1038/nature11162 22699612 PMC3376392

[140] Ali A, Shah L, Rahman S, Riaz MW, Yahya M, Xu YJ, et al Plant defense mechanism and current understanding of salicylic acid and NPRs in activating SAR. Physiological and Molecular Plant Pathology. 2018;104:15–22. 10.1016/j.pmpp.2018.08.001

[141] Blanco F, Salinas P, Cecchini NM, Jordana X, Van Hummelen P, Alvarez ME, et al Early genomic responses to salicylic acid in *Arabidopsis*. Plant Molecular Biology. 2009;70(1-2):79–102. 10.1007/s11103-009-9458-1 19199050

[142] Wang D, Amornsiripanitch N, Dong X. A genomic approach to identify regulatory nodes in the transcriptional network of systemic acquired resistance in plants. PLoS Pathogens. 2006;2(11). 10.1371/journal.ppat.0020123 PMC1635530 17096590

[143] Li Y, Huang F, Lu Y, Shi Y, Zhang M, Fan J, et al Mechanism of plant–microbe interaction and its utilization in disease-resistance breeding for modern agriculture. Physiological and Molecular Plant Pathology. 2013;83:51–58. 10.1016/j.pmpp.2013.05.001

[144] Xu G, Greene GH, Yoo H, Liu L, Marqués J, Motley J, et al Global translational reprogramming is a fundamental layer of immune regulation in plants. Nature. 2017;545(7655):487–490. 10.1038/nature22371 28514447 PMC5485861

[145] Xu G, Yuan M, Ai C, Liu L, Zhuang E, Karapetyan S, et al uORF-mediated translation allows engineered plant disease resistance without fitness costs. Nature. 2017;545(7655):491–494. 10.1038/nature22372 28514448 PMC5532539

[146] Park SW, Kaimoyo E, Kumar D, Mosher S, Klessig DF. Methyl salicylate is a critical mobile signal for plant systemic acquired resistance. Science. 2007;318(5847):113–116. 10.1126/science.1147113 17916738

[147] Baldwin IT, Halitschke R, Paschold A, Von Dahl CC, Preston CA. Volatile signaling in plant-plant interactions: “talking trees” in the genomics era. Science. 2006;311(5762):812–815. 10.1126/science.1118446 16469918

[148] Holopainen JK, Blande JD. Molecular plant volatile communication In: Sensing in nature. Springer; 2012 p. 17–31. 10.1007/978-1-4614-1704-0_2 22399393

[149] Filgueiras CC, Willett DS, Pereira RV, Junior AM, Pareja M, Duncan LW. Eliciting maize defense pathways aboveground attracts belowground biocontrol agents. Scientific Reports. 2016;6:36484 10.1038/srep36484 27811992 PMC5095600

[150] Filgueiras CC, Willett DS, Junior AM, Pareja M, El Borai F, Dickson DW, et al Stimulation of the salicylic acid pathway aboveground recruits entomopathogenic nematodes belowground. PloS One. 2016;11(5). 10.1371/journal.pone.0154712 27136916 PMC4854467

[151] Filgueiras CC, Willett DS, Pereira RV, Sabino PHdS, Junior AM, Pareja M, et al Parameters affecting plant defense pathway mediated recruitment of entomopathogenic nematodes. Biocontrol Science and Technology. 2017;27(7):833–843. 10.1080/09583157.2017.1349874

[152] Kokaly RF, Asner GP, Ollinger SV, Martin ME, Wessman CA. Characterizing canopy biochemistry from imaging spectroscopy and its application to ecosystem studies. Remote Sensing of Environment. 2009;113:S78–S91. 10.1016/j.rse.2008.10.018

[153] Thulin S, Hill MJ, Held A, Jones S, Woodgate P. Predicting levels of crude protein, digestibility, lignin and cellulose in temperate pastures using hyperspectral image data. American Journal of Plant Sciences. 2014;2014.

[154] Yendrek CR, Tomaz T, Montes CM, Cao Y, Morse AM, Brown PJ, et al High-throughput phenotyping of maize leaf physiological and biochemical traits using hyperspectral reflectance. Plant Physiology. 2017;173(1):614–626. 10.1104/pp.16.01447 28049858 PMC5210743

[155] Barth A. The infrared absorption of amino acid side chains. Progress in biophysics and molecular biology. 2000;74(3-5):141–173. 10.1016/S0079-6107(00)00021-3 11226511

[156] Ni J, Tian Y, Yao X, Zhu Y, Cao W. Application of monitoring system about plant growth information based on spectroscopy technique. In: PIAGENG 2010: Photonics and Imaging for Agricultural Engineering. vol. 7752. International Society for Optics and Photonics; 2011. p. 77521E.

[157] Martins RC, Magalhães S, Jorge P, Barroso T, Santos F. Metbots: Metabolomics Robots for Precision Viticulture. In: EPIA Conference on Artificial Intelligence. Springer; 2019. p. 156–166.

[158] Vergara Díaz O, et al. High-throughput phenotyping in cereals and implications in plant ecophysiology = Fenotipat de camp d’alt rendiment i implicacions en l’ecofisiologia vegetal. Universitat de Barcelona; 2019.

[159] Carter GA, Knapp AK. Leaf optical properties in higher plants: linking spectral characteristics to stress and chlorophyll concentration. American Journal of Botany. 2001;88(4):677–684. 10.2307/2657068 11302854

[160] Chandrashekara C, Bhatt J, Kumar R, Chandrashekara K. Supressive soils in plant disease management Eco-Friendly Innovative Approaches in Plant Disease Management, ed A Singh (New Delhi: International Book Distributors). 2012; p. 241–256.

[161] Pascale A, Proietti S, Pantelides IS, Stringlis IA. Modulation of the root microbiome by plant molecules: the basis for targeted disease suppression and plant growth promotion. Frontiers in Plant Science. 2020;10:1741 10.3389/fpls.2019.01741 32038698 PMC6992662

[162] Schlatter D, Kinkel L, Thomashow L, Weller D, Paulitz T. Disease suppressive soils: new insights from the soil microbiome. Phytopathology. 2017;107(11):1284–1297. 10.1094/PHYTO-03-17-0111-RVW 28650266

[163] Hennessy RC, Glaring MA, Olsson S, Stougaard P. Transcriptomic profiling of microbe–microbe interactions reveals the specific response of the biocontrol strain *P. fluorescens* In5 to the phytopathogen *Rhizoctonia solani*. BMC research notes. 2017;10(1):376 10.1186/s13104-017-2704-8 28807055 PMC5557065

[164] Castrillo G, Teixeira PJPL, Paredes SH, Law TF, de Lorenzo L, Feltcher ME, et al Root microbiota drive direct integration of phosphate stress and immunity. Nature. 2017;543(7646):513–518. 10.1038/nature21417 28297714 PMC5364063

[165] Massart S, Martinez-Medina M, Jijakli MH. Biological control in the microbiome era: challenges and opportunities. Biological Control. 2015;89:98–108. 10.1016/j.biocontrol.2015.06.003

[166] Köhl J, Kolnaar R, Ravensberg WJ. Mode of action of microbial biological control agents against plant diseases: relevance beyond efficacy. Frontiers in Plant Science. 2019;10:845 10.3389/fpls.2019.00845 31379891 PMC6658832

[167] Vukicevich E, Lowery T, Bowen P, Úrbez-Torres JR, Hart M. Cover crops to increase soil microbial diversity and mitigate decline in perennial agriculture. A review. Agronomy for Sustainable Development. 2016;36(3):48 10.1007/s13593-016-0385-7

[168] Bonanomi G, Lorito M, Vinale F, Woo SL. Organic amendments, beneficial microbes, and soil microbiota: toward a unified framework for disease suppression. Annual Review of Phytopathology. 2018;56:1–20. 10.1146/annurev-phyto-080615-100046 29768137

[169] van Bruggen AH, Gamliel A, Finckh MR. Plant disease management in organic farming systems. Pest Management Science. 2016;72(1):30–44. 10.1002/ps.4145 26331771

[170] Hiddink GA, Termorshuizen AJ, van Bruggen AH. Mixed cropping and suppression of soilborne diseases In: Genetic Engineering, biofertilisation, soil quality and organic farming. Springer; 2010 p. 119–146.

[171] Khare E, Mishra J, Arora N. Multifaceted Interactions Between Endophytes and Plant: Developments and Prospects. Frontiers in Microbiology. 2018;9:2732 10.3389/fmicb.2018.02732 30498482 PMC6249440

[172] Ahmad I, del Mar Jiménez-Gasco M, Luthe DS, Shakeel SN, Barbercheck ME. Endophytic *Metarhizium robertsii* promotes maize growth, suppresses insect growth, and alters plant defense gene expression. Biological Control. 2020; p. 104167 10.1016/j.biocontrol.2019.104167

[173] Kusajima M, Shima S, Fujita M, Minamisawa K, Che FS, Yamakawa H, et al Involvement of ethylene signaling in *Azospirillum sp*. B510-induced disease resistance in rice. Bioscience, biotechnology, and biochemistry. 2018;82(9):1522–1526. 10.1080/09168451.2018.1480350 29847205

[174] Sheibani-Tezerji R, Rattei T, Sessitsch A, Trognitz F, Mitter B. Transcriptome profiling of the endophyte *Burkholderia phytofirmans* PsJN indicates sensing of the plant environment and drought stress. MBio. 2015;6(5). 10.1128/mBio.00621-15 26350963 PMC4600099

[175] Jia Q, Qu J, Mu H, Sun H, Wu C. Foliar endophytic fungi: diversity in species and functions in forest ecosystems. Symbiosis. 2020; p. 1–30.

[176] Harrison JG, Griffin EA. The diversity and distribution of endophytes across biomes, plant phylogeny and host tissues: how far have we come and where do we go from here? Environmental Microbiology. 2020 10.1111/1462-2920.14968 PMC7679042 32115818

[177] Meireles JE, Cavender-Bares J, Townsend PA, Ustin S, Gamon JA, Schweiger AK, et al Leaf reflectance spectra capture the evolutionary history of seed plants. New Phytologist. 2020 10.1111/nph.16771 32579721 PMC7540507

[178] Galla SJ, Buckley TR, Elshire R, Hale ML, Knapp M, McCallum J, et al Building strong relationships between conservation genetics and primary industry leads to mutually beneficial genomic advances. Molecular ecology. 2016;25(21):5267–5281. 10.1111/mec.13837 27641156

[179] Talbot JM, Bruns TD, Taylor JW, Smith DP, Branco S, Glassman SI, et al Endemism and functional convergence across the North American soil mycobiome. Proceedings of the National Academy of Sciences. 2014;111(17):6341–6346. 10.1073/pnas.1402584111 PMC4035912 24733885

[180] Fierer N, Leff JW, Adams BJ, Nielsen UN, Bates ST, Lauber CL, et al Cross-biome metagenomic analyses of soil microbial communities and their functional attributes. Proceedings of the National Academy of Sciences. 2012;109(52):21390–21395. 10.1073/pnas.1215210110 PMC3535587 23236140

[181] R Cole’s J, Wang Q, Fish J, Chai B, Mcgarrell D, Sun Y, et al Ribosomal DATABASE PROject: data and tools for high throughput rRNA analysis. Nucleic acids research. 2013;42 10.1093/nar/gkt1244 24288368 PMC3965039

[182] Quast C, Pruesse E, Yilmaz P, Gerken J, Schweer T, Yarza P, et al The SILVA ribosomal RNA gene database project: Improved data processing and web-based tools. Nucleic acids research. 2012;41 10.1093/nar/gks1219 PMC3531112 23193283

[183] Mcdonald D, Price M, Goodrich J, Nawrocki E, DeSantis T, Probst A, et al An improved GreenGenes taxonomy with explicit ranks for ecological and evolutionary analyses of Bacteria and Archaea. The ISME journal. 2011;6:610–8. 10.1038/ismej.2011.139 22134646 PMC3280142

[184] Nilsson RH, Larsson KH, Taylor A, Bengtsson-Palme J, Jeppesen T, Schigel D, et al The UNITE database for molecular identification of fungi: handling dark taxa and parallel taxonomic classifications. Nucleic Acids Research. 2019;47:D259–D264. 10.1093/nar/gky1022 30371820 PMC6324048

[185] Nguyen N, Song Z, Bates S, Branco S, Tedersoo L, Menke J, et al FUNGuild: An open annotation tool for parsing fungal community datasets by ecological guild. Fungal Ecology. 2015;20.

[186] Zanne AE, Abarenkov K, Afkhami ME, Aguilar-Trigueros CA, Bates S, Bhatnagar JM, et al Fungal functional ecology: bringing a trait-based approach to plant-associated fungi. Biological Reviews. 2020;95(2):409–433. 10.1111/brv.12570 31763752

[187] Horai H, Arita M, Kanaya S, Nihei Y, Ikeda T, Suwa K, et al MassBank: a public repository for sharing mass spectral data for life sciences. Journal of Mass Spectrometry. 2010;45(7):703–714. 10.1002/jms.1777 20623627

[188] Sud M, Fahy E, Cotter D, Azam K, Vadivelu I, Burant C, et al Metabolomics Workbench: An international repository for metabolomics data and metadata, metabolite standards, protocols, tutorials and training, and analysis tools. Nucleic Acids Research. 2016;44(D1):D463–D470. 10.1093/nar/gkv1042 26467476 PMC4702780

[189] Johnson SR, Lange BM. Open-access metabolomics databases for natural product research: present capabilities and future potential. Frontiers in Bioengineering and Biotechnology. 2015;3:22 10.3389/fbioe.2015.00022 25789275 PMC4349186

[190] Sarrocco S, Herrera-Estrella A, Collinge DB. Plant Disease Management in the Post-genomic Era: From Functional Genomics to Genome Editing. Frontiers in Microbiology. 2020;11:107 10.3389/fmicb.2020.00107 32117135 PMC7010928

